# Transcultural adaptation and validation of familial satisfaction in the intensive care unit in Korea: preliminary study

**DOI:** 10.1186/2197-425X-3-S1-A654

**Published:** 2015-10-01

**Authors:** J Shin, Y Kim, H Lee, J Lee, KS Kim, YJ Cho, YH Jo, H Rhu, KS Kim, SM Lee, J Min, G Park, J Yoon, SI Park, YJ Lee

**Affiliations:** Seoul National University Bundang Hospital, Seongnam, Republic of Korea; Seoul National University Hospital, Seoul, Republic of Korea

## Introduction

Lately, in North America, questionnaires have been developed and validated, which were to assess family needs and satisfaction with care in the intensive care unit. One of the most widely used one is FS-ICU-24 survey.

## Objectives

The purpose of this study is to prove the Korean version of FS-ICU-24 survey.

## Methods

The study was performed in the medical, surgical, and emergency ICU of Seoul National University Bundang Hospital and Seoul National University Hospital. Relatives of all patients with a length of stay of 48 hours or longer were eligible for the study. And the validation included feasibility, construct validity, internal consistency, reliability and sensitivity. The survey consisted of 24 items and two categories: (i) satisfaction with care (14 items) and (ii) satisfaction with decision making (10 items).

## Results

The translated form was distributed to 81 family members. The response rate was 61.7% and 97.9% of questions in returned forms were answered. Compared with a Visual Analogue Scale, the construct validity was good for the total survey satisfaction (Spearman p = 0.795). A Cronbach a coefficient for satisfaction with care subscale was 0.953; for satisfaction with decision making subscale was 0.886. The total FS-ICU-24 survey mean score was 78.48 ± 15.56. In this study, the responders were most satisfied with having adequate time to have their concerns addressed and questions answered (89.8 ± 30.58) and least satisfied with the atmosphere of the ICU waiting room (41.46 ± 36.91).

## Conclusions

A cross-cultural adaptation of FS-ICU-24 into Korean version can be validated enough and will be acquired reliability.

